# TGL / HDL - C ratio and non-HDL-C - prognostic index in chronic kidney disease

**DOI:** 10.6026/973206300220875

**Published:** 2026-02-28

**Authors:** Siva I, G. Chitra Siva Sankari, Vishaalpalaniswamy Ramaswamy, Amuthavalli Vasudevan, Pramila K.

**Affiliations:** 1 Institute of Biochemistry, Madras Medical College & Rajiv Gandhi Government General Hospital, Chennai, Tamil Nadu MGR University Chennai, India; 2 Department of Biochemistry, Government Theni Medical College & Hospital, Theni, Tamil Nadu MGR University Chennai, India

**Keywords:** Chronic kidney disease (CKD), Dyslipidemia

## Abstract

Dyslipidemia, a major risk factor for chronic kidney disease (CKD) affects more than ten percent of the general population. Dyslipidemia in CKD, if left untreated, will result in end stage renal disease. So, the relationship between atherogenic lipid profile ratios and the assessment of CKD severity is a potential area still to be explored. In assessing the severity of CKD, these ratios have higher prognostic value than individual parameters alone. Therefore, it is of interest to evaluate the role of TGL/HDL- c ratio and NON- HDL-c levels in assessing the disease severity in CKD. Cross-sectional study with one hundred and thirteen CKD patients divided into two groups based-on e-GFR levels and CKD-EPIDEMIOLOGY Staging. Quantitative measurements of lipid profile parameters (total cholesterol, triglycerides, HDL-c) done by enzymatic colorimetric method and renal function test by spectrophotometric method. TGL: HDL-c and NON- HDL-c levels were calculated, Statistical data analyzed using SPSS version software 16.0 showed TGL / HDL- c ratio and NON-HDL-c values has significant correlation with CKD staging and found to highly significant (p <0.001). TGL / HDL-c ratio and NON-HDL-c value can be used as a prognostic index in CKD patients.

## Background:

Chronic kidney disease (CKD) affecting more than eight hundred million population worldwide, is defined as GFR value less than 60 ml/min/1.73 m^2^ and decline in renal structure or function lasting over three months [1, 2]. CKD affects more than ten percent of the global population [3]. In India, prevalence of CKD is eight percent between 2018-2023 [4]. Dyslipidemia - elevated triglycerides (TGL), total cholesterol (TCL) and low-density lipoprotein cholesterol (LDL-c) along with reduced high density lipoprotein cholesterol (HDL-c) leads to inflammation, endothelial dysfunction and structural damage [5, 6]. These disruptions form the basis of the lipid nephrotoxicity [7]. Excessive triglycerides and cholesterol can injure podocytes, influence mesangial activity and contribute to glomerulosclerosis, thereby accelerating the severity of CKD [8] TGL/HDL-c ratio and NON-HDL-c levels, strongly correlates with cardiovascular risk and kidney dysfunction [9, 10, 11-12]. Assessment of dyslipidaemia is essential for identifying the disease severity in CKD [13]. Therefore, it is of interest to evaluate the role of TGL/HDL-c ratio and NON-HDL-c levels in assessing the disease severity in CKD.

## Materials and Methods:

This cross-sectional observational study was conducted during the period of July to September 2024 in Institute of Biochemistry and Institute of Nephrology in Madras Medical College and Rajiv Gandhi Government General Hospital, Chennai.

## Study population:

The study was conducted after getting ethical committee approval and comprised of one hundred and thirteen patients (seventy-one males, forty-two females) divided into groups based on e-GFR levels and CKD - EPI staging. Group one with stages (one and two) which includes thirty-eight males and twenty-two females comprises of sixty patients. Group two with stages (three, four and five) which includes thirty-three males and twenty females comprises of fifty-three patients. Patients were selected from confirmed CKD cases based on e-GFR levels and KDIGO GUIDELINES admitted and treated in inpatient ward and outpatient department from Institute of Nephrology in Rajiv Gandhi Government General Hospital, Chennai. Patients who were diagnosed as a case of CKD as per on e-GFR levels and KDIGO GUIDELINES were included for the study and exclusion criteria includes patients with co morbidities like diabetes mellitus, auto immune diseases. Patients taking lipid lowering drugs, antioxidants, hormonal therapy.

## Sample collection:

After getting written informed consent, five mL of venous blood was drawn from antecubital vein of patients and collected in a plain vacutainer tube under aseptic precautions and serum separated by centrifugation at three thousand revolutions per minute (rpm) at fifteen minutes. Analytes were estimated using Roche automated clinical chemistry analyzer. Quantitative measurements of lipid profile parameters (total cholesterol, triglycerides and HDL-c) done by enzymatic colorimetric method and renal function test done by spectrophotometric method and complete blood count done by automated cell counter.

## Statistical analysis:

Statistical data was analyzed using SPSS (statistical package for social science) version software 16.0. Mean and standard deviation were calculated. Calculations of p values were done and the values of significance were determined. A p value less than of 0.001was considered to be highly significant and less than of 0.05 was considered to be significant. Receiver operating characteristic (ROC) curve was done to assess the sensitivity and specificity of TGL: HDL-c and NON -HDL-c. Area under curve (AUC) was useful to find out the expected cases of CKD.

## Results and Discussion:

Table 1 shows the mean and p values of TGL: HDL-c ratio, NON-HDL-c, TCL, TGL, HDL and Hb in group one (stages one and two) and group two (stages three, four and five) CKD patients. It reveals that TGL: HDL-c ratio, NON-HDL-c and TCL have a highly significant (< 0.001) p-value and TGL has significant p value (0.005). Figure 1A reveals the mean concentration of TGL: HDL-c on Y axis and two different study groups (group1[CKD stages 1, 2]) and (group2[CKD stages 3, 4 and 5]) on X axis. Inference - Group 2 has higher TGL: HDL-c ratio than group. Figure 1B; reveals the mean concentration of NON- HDL-c(mg/dL) on Y axis and two different study groups (group1[CKD stages 77 1,2]) and (group2[CKD stages3, 4 and 5]) on X axis. Inference - Group 2 has higher NON - HDL c value than Group 1. Figure 2A and Table 2 reveals that area under curve for TGL: HDL-c ratio is 0.691 and ROC curve showed sensitivity - 54 % and specificity - 82%. Figure 2B and Table 3 reveals that area under curve for NON -HDL-c is 0.717 and ROC curve showed that sensitivity - 58% and specificity - 77%. Chronic kidney disease is a progressive condition which leads to nephron damage followed by compensatory hyperfiltration in the remaining nephrons, leading to structural and functional deterioration of kidney [14]. Dyslipidemia which occurs in CKD initiates pathological processes such as endothelial dysfunction, oxidative injury and glomerular structural distortion [15]. This study was conducted to evaluate the role of dyslipidemia in CKD, five such parameters were selected which includes TGL, HDL-c, TGL/HDL-c ratio, NON-HDL-c and TCL TGL, elevated in early stages of CKD. [Reference range (40-120 mg/dL) [16]. Elevated TGL levels ([160 ± 32.7] mg/dL), in group one and ([196 ± 82] mg/dL), in group two and found to be significant in study groups (p value - 0.002) as seen in Table 1. The increase in triglyceride levels in CKD reflects the alteration of lipoprotein metabolism observed by study conducted by Bhattacharjee *et al.* [17]. This is due to reduced activity of lipoprotein lipase and hepatic triglyceride lipase [18]. Impaired clearance of VLDL-c and chylomicron results in formation of triglyceride-rich lipoprotein particles [19]. These particles have a high risk to cause vascular injury in kidney [20]. Table 1 revels that HDL-c levels did not show statistical significance with CKD groups. In CKD, HDL-c maturation and functions are compromised, including the formation of nascent HDL from apo A-I, ABCA1-mediated cholesterol efflux and LCAT-dependent esterification process required for HDL to mature into larger, more cholesterol-rich forms [21, 22- 23]. Despite the absence of numerical difference between groups, HDL-c impairment remains clinically relevant in CKD patients. TGL / HDL-c ratio has highly significant association (p value - <0.001) with values of (4.29 ± 1.24) in Group one and (5.88 ± 2.57) in Group two, as in Table 1. This correlates with previous study conducted by Kang *et al.* [24]. This ratio (Reference value - <3) reflects the combined impact of elevated triglycerides and functionally impaired HDL-c, both of which contribute to a pro-atherogenic metabolic profile. Elevated TGL / HDL-c ratio is inducing glomerulosclerosis and mesangial cells proliferation leads to worsening of CKD [25, 26, 27-28]. Figure 1A reveals that TGL/ HDL-c ratio is higher in group two than group one, which implies that the ratio increased with the progression of CKD. Table 2 and Figure 2A revels that AUC for TGL: HDL-c ratio is 0.691 and ROC curve showed that the ratio had a sensitivity of 54% and specificity of 82% NON-HDL-c, increases as CKD stage advances as seen Table 1, it includes all atherogenic lipoproteins (reference value -< 130mg/dL). In this study, NON - HDL-c value showed highly significant association (p value -<0.001) with CKD progression, with values of ([124 ± 33.8] mg/dL), in Group one and ([163 ± 64.3] mg/ dL) in Group two. Figure 1B reveals NON-HDL- c value is higher in later stages compared to earlier stages indicating the disease progression. Study conducted by Wen *et al.* [29] has correlated elevated NON-HDL-c levels with adverse renal outcomes [30]. Table 3 and Figure 2B reveals that AUC for NON -HDL-c is 0.717, sensitivity of 58% and specificity of 77%. TCL levels were increased with CKD stage contributing of lipid dysregulation reference range - 200 mg/dL. In this study, TCL elevated in study groups, with levels of ([163 ± 38.6] mg/dL), in Group one and ([199 ± 65.1] mg/dL), in Group two, with a highly significant p value (<0. 001).as seen in Table 1. Higher TCL levels in early and intermediate stages indicate the renal impairment associated with impaired lipoprotein clearance [31].

## Conclusion:

Higher levels of TGL / HDL-c ratio and NON -HDL-c value indicate that patient is advancing to later stages of CKD. Calculating these indices from lipid profile is an inexpensive and easily available tool in identifying disease progression even in primary health care set up. Thus, we show that TGL / HDL-c ratio and NON -HDL-c value can be used as a prognostic index in identifying the disease severity of CKD.

## Scope of the study:

This study can be done as a case control study and age and sex matched healthy individuals can be compared.

## Limitations of the study:

[1] Sample size

[2] Want of follow up

## Figures and Tables

**Figure 1A:**
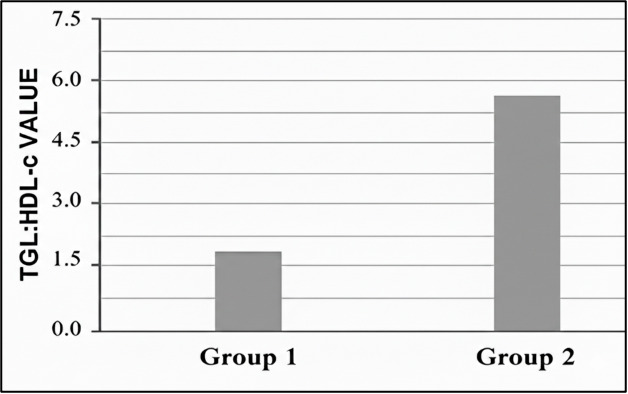
Mean concentrations of TGL: HDL-c ratio value among study groups

**Figure 1B:**
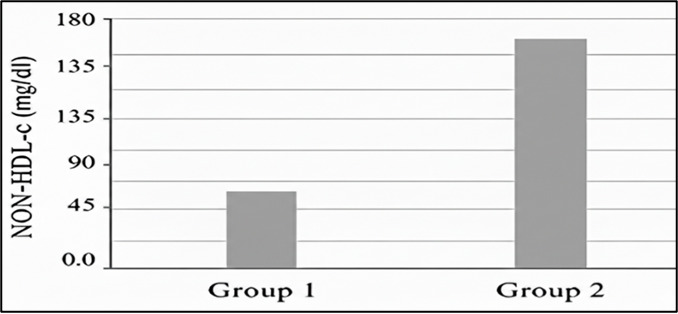
Mean concentrations of non - HDL-c value among study groups

**Figure 2A:**
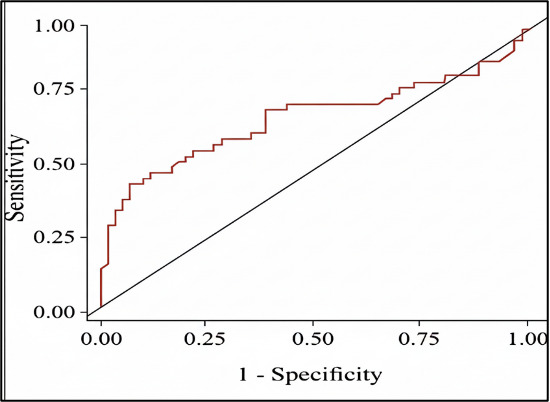
Receiver operating characteristic curve TGL: HDL-c VS CKD stage

**Figure 2B:**
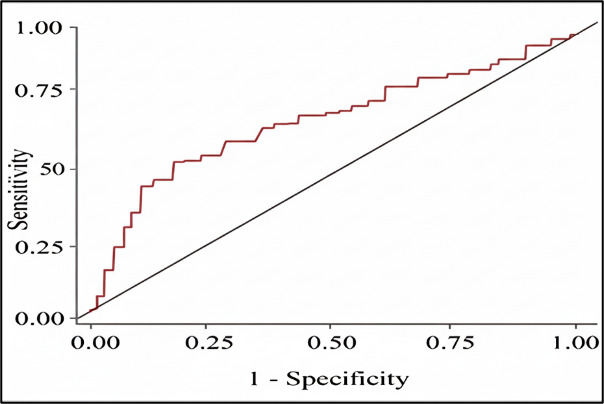
Receiver operating characteristic curve NON- HDL -c VS CKD stage

**Table 1:** Laboratory findings of lipid profile and other parameters in group 1 (stages 1 and 2) and group 2 (stages 3, 4 and 5) CKD patients

**Parameter**	**Group 1**	**Group 2**	**P-value**
No of participants	60	53	-
Age	46	48.6	-
Sex(M/F)	38/22	33/20	-
**Biochemical parameters**			
HDL-c(mg/dL)	39.1 ± 11.4	35.6 ± 10.7	0.099
TGL (mg/dL)	160 ± 32.7	196 ± 82	0.002*
TGL: HDL-c	4.29 ± 1.24	5.88 ± 2.57	<0.001*
TCL (mg/dL)	163 ± 38.6	199 ± 65.1	<0.001*
NON-HDL- c	124 ± 33.8	163 ± 64.3	<0.001*
**c (mg/dL)**			
Hb (g/dL)	10.5 ± 1.81	10 ± 1.72	0.135
*-highly significant

**Table 2:** Area under curve value (AUC) for TGL: HDL-c variable

**Test variable: TGL;HDL-c**				
**Area**	**Std error**	**Asymptotic sig**	**Asymptotic 95% confidence interval**	
0.691	0.117	0.433	Upper level	Lower level
			0.51	0.187

**Table 3:** Area under curve value (AUC) for NON -HDL-c variable

**Test variable: NON -HDL-c**				
**Area**	**Std error**	**Asymptotic sig**	**Asymptotic 95% confidence interval**	
0.717	0.005	0.019	Upper level	Lower level
			0.438	0.096
